# Systematic implementation of rapidplan for prostate cancer: toward a unified knowledge-based planning model

**DOI:** 10.1038/s41598-025-08553-7

**Published:** 2025-07-01

**Authors:** Ahmed Hadj Henni, Asma Hamouali, Alexandre Marque, Ilias Arhoun

**Affiliations:** 1 Centre Frédéric Joliot, Rouen, France; 2 Clinique Saint Hilaire, Rouen, France

**Keywords:** RapidPlan™, Knowledge-based planning, Prostate cancer treatment, Dose–volume constraints, Treatment planning, Biophysics, Cancer, Physics

## Abstract

This study presents a comprehensive methodology for implementing a unified knowledge-based planning model for RapidPlan^™^ (RP) to manage all 11 prostate cancer prescriptions used at our institution. Several RP configurations were evaluated to address different clinical scenarios. The initial models RP_46 and RP_30 involved the prostate, seminal vesicles, and lymph nodes treated with 46 Gy in 23 fractions and the prostate alone treated with 30 Gy in 15 fractions, respectively. These models were progressively expanded to incorporate all sequential boost treatment plans (RP_46 + 30), including those targeting the prostate bed (RP_Seq). Simultaneous integrated boost prescriptions were used to train the RP_SIB model, which was subsequently combined with the RP_Seq model to form the unified RP_UNI model. Each configuration was compared with the manual method using a cohort of 10–25 patients. All the models produced treatment plans that met the clinical requirements. An overall analysis revealed that the RP_UNI model significantly reduced the 45 Gy and 15 Gy volumes (cm^3^) in the peritoneal cavity by approximately 18%. The RP_UNI model was chosen for clinical implementation owing to its broader applicability compared with the other models, offering a 66% reduction in planning time with respect to the manual method. The unified model, derived from simpler RP configurations, successfully integrated all 11 prostate cancer prescriptions used at our institution. This model performed efficiently regardless of the complexity of the target volumes or whether the irradiation technique was a sequential or simultaneous integrated boost.

## Introduction

Intensity-modulated radiation therapy (IMRT) has become the standard treatment modality for numerous tumor types. Since the 1980s and 1990s, advances in accelerator technology have introduced degrees of freedom^1^ that enable high conformal dose delivery to the planning target volume (PTV) while minimizing exposure of surrounding organs at risk (OARs). In volumetric modulated arc therapy (VMAT), dose modulation is achieved through the coordinated use of gantry rotation, jaw and multileaf collimator movements, and variable dose rates.

Treatment planning relies on a complex inverse optimization process, with plan quality heavily influenced by human operators. Key factors such as beam configuration, selection of dose objectives, and application of optimization constraints significantly affect the outcome^2^. This manual process is labor intensive, requires extensive operator training, and does not inherently ensure qualitatively optimal plans, even when regulatory dose constraints are met^3,4^. Suboptimal planning has adversely impacted therapeutic outcomes^5,6^.

These challenges underscore the need for refined optimization processes. Data-driven approaches have emerged, leveraging the planning knowledge accumulated in radiotherapy centers to predict outcomes for new cases. One such approach, knowledge-based planning (KBP), was introduced approximately 15 years ago^7,8^. KBP uses anatomical and geometric features—such as distances between target structures and overlap between the OAR and PTV—to construct mathematical models. These models predict key dosimetric parameters for new cases, including dose–volume histograms (DVHs) and spatial dose distributions.

Since 2008, numerous studies^7^ have demonstrated that KBP methods can standardize and enhance planning practices, improve the quality of plans submitted for clinical validation, and provide effective training tools for less experienced operators.

Various research teams have developed “in-house” KBP^9–11^, among which the work of Moore et al.^12^ is particularly notable. A commercial automated planning solution based on the KBP methodology, RapidPlan™ (RP), was introduced in 2014 and integrated into the Eclipse treatment planning system (TPS) developed by Varian Medical Systems^13^.

Several studies have demonstrated the utility of RP across a broad spectrum of cancer sites. Fogliatia et al., recognized for their extensive contributions in this domain, highlighted the applicability of RP in the treatment of lung^14^, prostate^15^, liver^14^, esophagus^16^, head and neck^17^, and breast^18^ cancers. Additional studies have explored its use in other anatomical sites^19,20^.

However, the implementation of RP is challenging. The careful selection of training datasets is critical for developing high-performance models. Studies by Delaney et al.^21^ and Alpuche Aviles et al.^22^ have examined the impact of atypical plans (outliers) on dosimetric outcomes, highlighting the complexity of this step. Moreover, during the implementation of RP for prostate cancer treatment, several issues emerged that were either unaddressed by the manufacturer or inadequately covered in the literature, complicating its clinical adoption such as :

- Accommodating the diversity of clinical practices: Eleven distinct medical prescriptions (Table 1), including those for the prostate bed, were identified across the protocol of the center. It is thus necessary to design RP templates that address this prescription variability.
- Standardizing prescription methods: Sequential boost and simultaneous integrated boost (SIB) approaches are fundamentally different. The proposed methodology should determine whether these approaches can be unified within a single model or whether separate models should be developed.
- Target volume complexity: Variations in target volume complexity— ranging from pelvic volumes that include lymph nodes to isolated prostate targets—require the model to be adaptable to accurately reflect these different clinical scenarios.

Therefore, this study aims to establish a systematic methodology for developing one or more models tailored to prostate cancer treatment, addressing all these key challenges.

The proposed methodology refines two initial simplified models through a series of distinct, progressive steps. Each iteration is compared with alternative configurations to optimize the performance. No prior methodology has successfully integrated 11 diverse prostate cancer prescriptions into a unified model that addresses the varying complexities of target volumes.

The proposed methodology, which focuses on prostate cancer, can be adapted to other anatomical sites facing similar challenges.

## Methods

### Global overview and patient selection

Dosimetric data from 226 prostate cancer patients treated at our institution between November 2021 and March 2024 were used to develop, compare, and validate the models described in this study. This study involved a retrospective analysis of fully anonymized data; hence, informed consent was waived.

Patients were positioned supine using standard immobilization devices. Computed Tomography (CT) images (Somatom Definition AS20 RT, Siemens©) were acquired according to a standardized pelvic imaging protocol ensuring an empty rectum and full bladder. A slice thickness of 1.5 mm was employed to ensure high-resolution imaging. For the bladder, the applied tolerance was between 150 and 350 cm³. A check was then carried out at the treatment room prior to irradiation. For the rectum, the radiation therapists identified the most unfavorable slice on both CT and CBCT (Cone Beam Computed Tomography) scans, i.e. the one where the rectum was widest in the antero-posterior direction. On this slice, they measured the antero-posterior distance, which should not exceed 4 cm.

The irradiation modalities and prescriptions recorded at our center are summarized in Table 1. A total of 11 distinct prescriptions were included, encompassing treatments for the prostate alone or in combination with seminal vesicles (SV), with or without pelvic lymph node (LN) or nodes boost (Ns) involvement. The target volumes were treated with one or more plans (≤ 3) using either sequential boost or SIB techniques. Prostate bed irradiation plans were also incorporated into this study.

**Table 1:** Description of the 11 prescriptions for the treatment of prostate cancer using sequential or simultaneous integrated boost techniques. SV: seminal vesicles, LN: pelvic lymph nodes, ns: Nodes.

Technique	Prescription (*P*)		Number of plans	Localisation/Prescription details
**Sequential**	**P1**		2	**P1.1 =** Prostate + SV + LN : 46 Gy in 23 fractions
				**P1.2 =** Prostate : 30 Gy in 15 fractions
	**P2**		2	**P2.1 =** Prostate bed + LN :46 Gy in 23 fractions
				**P2.2 =** Prostate bed : 20 Gy in 10 fractions
	**P3**		2	**P3.1 =** Prostate bed : 66 Gy in 33 fractions
				**P3.2 =** Prostate Boost : 4 Gy in 2 fractions
	**P4**		1	**P4 =** Prostate : 76 Gy in 38 fractions
	**P5**		1	**P5 =** Prostate bed : 66 Gy in 33 fractions
**Simultaneous Integrated Boost (SIB)**	**P6**		3	**P6.1 =** Ns Boost 58.8 Gy + Prostate 56 Gy + SV left or right + Ns 56 Gy + LN 50.4 Gy in 28 fractions
				**P6.2 =** Prostate + SV left or right : 4 Gy in 2 fractions
				**P6.3 =** Prostate : 16 Gy in 8 fractions
	**P7**		2	**P7.1 =** Prostate bed :50 Gy and LN :45 Gy in 25 fractions
				**P7.2 =** Prostate bed :16 Gy in 8 fraction
	**P8**		3	**P8.1 =** Prostate + SV : 50 Gy and LN :45 Gy and PTVnSIB: 56.25 Gy in 25 fractions
				**P8.2 =** Prostate : 26 Gy in 13 fractions
				**P8.3 =** Prostate Boost : 4 Gy in 2 fractions
	**P9**		2	**P9.1 =** Prostate + SV : 50 Gy and LN :45 Gy in 25 fractions
				**P9.2 =** Prostate : 26 Gy in 13 fractions
	**P10**		1	**P10 =** Prostate : 60 Gy and SV : 44 Gy in 20 fractions
	**P11**		2	**P11.1 =** Prostate bed and LN 46 Gy and Ns Boost : 48.3 Gy in 23 fractions
				**Plan 11.2 =** Prostate bed : 20 Gy and Ns Boost : 21 Gy in 10 fractions

This study compared several configurations to address the variability in medical prescriptions for prostate cancer. Two foundational models, RP_46 and RP_30, were created using treatment plans:

- RP_46: 46 Gy delivered in 23 fractions to the prostate, seminal vesicles, and lymph nodes.
- RP_30: 30 Gy delivered in 15 fractions solely to the prostate.

At the time of the initial implementation phase of the RP solution, this prescription was the most frequent.

These initial models were sequentially extended to include all sequential boost treatment plans, including those targeting the prostate bed (RP_Seq). Each stage of model development was evaluated against a cohort of patients and compared with the manual method (MM). Hence, the RP_46 + 30 model, which combines the RP_46 and RP_30 models, and the RP_Seq model, which includes plans with prostate bed irradiation, were created.

All SIB prescriptions were used to develop the RP_SIB model. These plans were subsequently added to the RP_Seq model to create a unified model, RP_UNI, designed to deliver high-quality treatment plans across all 11 medical prescriptions. Following the same approach, the RP_SIB and RP_UNI models were evaluated by comparing them to several manually created reference plans.

**Fig. 1:**
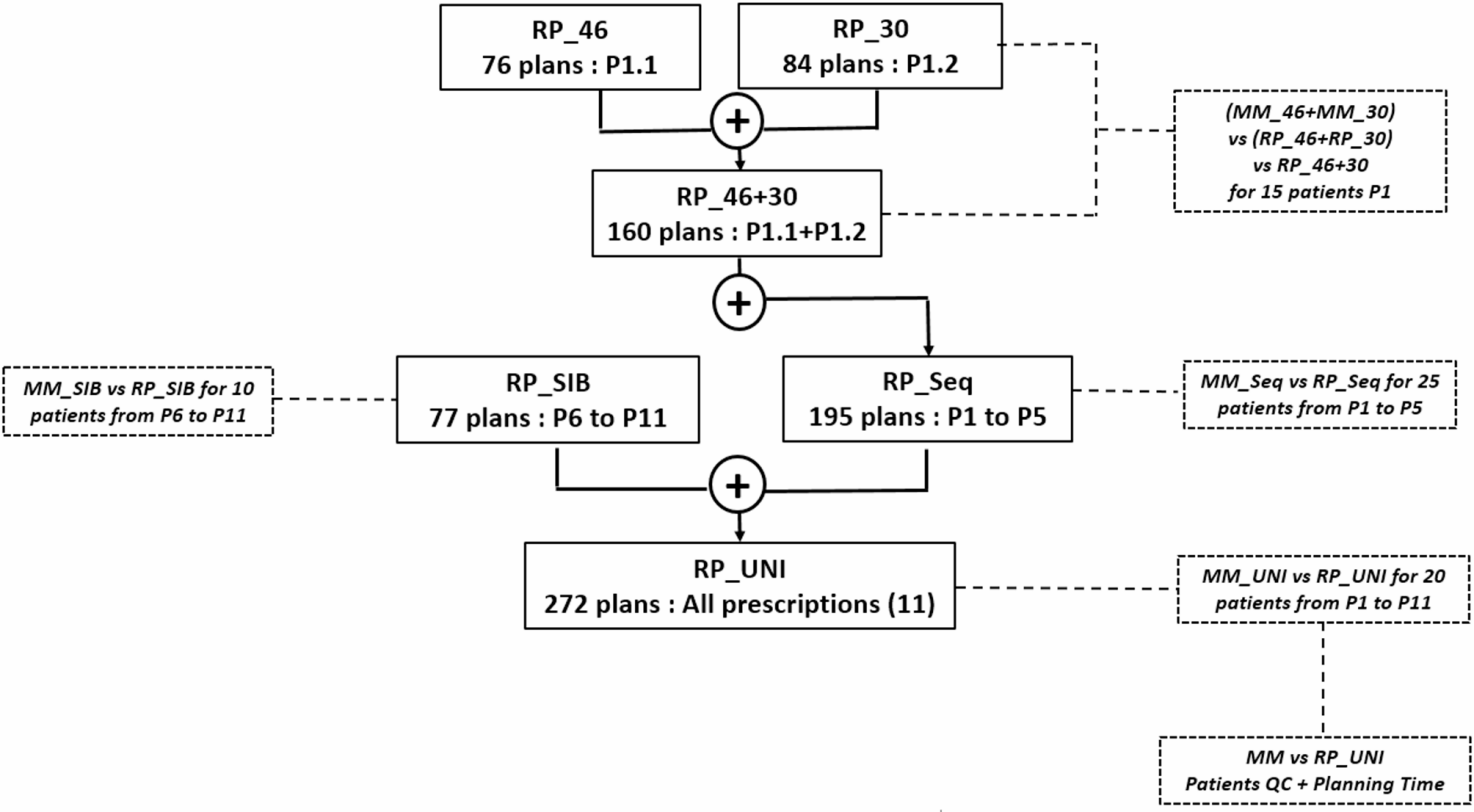
Workflow outlining the steps to develop the RapidPlan™ model for managing the 11 medical prescriptions used in clinical practice. Two initial models, RP_46 and RP_30, were progressively expanded to incorporate all sequential boost treatment plans (RP_46 + 30), including the prostate bed (RP_Seq). The model containing only integrated boost plans (RP_SIB) was combined with the RP_Seq model to create the RP_UNI model. Each step was validated through tests involving 10–25 patients external to the models and comparisons with results from manual planning.

Figure 1 illustrates the process of developing an RP model (RP_UNI), which integrates all medical prescriptions used at our center. Each comparison step was based on a cohort of 10–25 patients, which led to our RP_UNI model. A total of 70 patient cases containing between 1 and 3 treatment plans (Table 1) were used for validation. The aim was to find a trade-off between robustness of the results and pragmatism of clinical implementation. For example, Fogliata et al.^23^, in an iterative approach to improving their RP models, used a cohort of 20 patients to compare them.

Dosimetry completion times were recorded for RP_UNI and the MM in the latter set of measurements. Quality control outcomes based on an onboard portal imager (EPID, As1200) were evaluated and compared for these two planning methods. The mean number of MUs per plan obtained by the RP_UNI model were also compared to those obtained by the manual method.

### Treatment planning technique

All treatment plans were generated using the TPS Eclipse with an AAA 15.6 algorithm (Varian Medical Systems, Palo Alto, CA, USA) and VMAT. The operators used ballistics configurations consisting of 2–4 arcs with a 6 MV flattening filter (FF) or 6 MV free FF (FFF) at 600 MU/min, depending on the accelerator used (Truebeam© or Halcyon©). The number of arcs was determined by the complexity of the target volume and remained consistent across plans evaluated with different RP models.

A preliminary qualitative estimation of the RP plans was carried out by experienced physicists and physicians prior to the quantitative analysis. The DVH of the different structures were then used to conduct our quantitative study.

The plan validation criteria required at least 95% of the prescribed dose to cover the PTV (D95% > 95%) and a Dmax below 107%^24^. The Paddick conformity index (PCI)^25^ and homogeneity index (HI)^24^ were calculated for each plan. Conformity of the prescription dose to the target volume was considered acceptable for a PCI above the threshold value of 0.7. A homogeneous distribution in the PTV results in an HI close to 0. Dosimetric constraints for routine clinical practice were based on the cumulative doses of the patient’s treatment plans.

### Statistical analysis

All the RP models were constructed following the statistical guidelines established by the system manufacturer. Model creation involved analyzing graphs provided by the RP module (linear regressions, residuals, and dose–volume histograms) while identifying geometric or dosimetric outliers that could affect predictive performance. Statistical evaluation parameters, namely the coefficient of determination (R²) and mean chi-square (χ²), were required to establish equivalence across all the RP models. None of the RP models incorporated optimization volumes, such as rings or overlaps, which are commonly used in manual planning. The proposed methodology precluded planner intervention in the RP modeling process.

 Table 2 Summarizes the configuration of DVH constraints applied to PTVs and oars with identical definitions and priorities for all RP models. Briefly, the constraints can be “generated” from the model’s learning, based on the data used in the training sample. For a given case, depending on the presented geometry, the model automatically adjusts the constraints — whether in terms of volume, dose, or priority. Unlike manually defined and fixed constraints set by the operator for each case, the model operates autonomously and adapts to the specificity of each case. The lines(target) constraints correspond to a set of upper-type constraints that follow a decreasing slope across the entire DVH of the OAR. Due to the multiplicity of upper constraints generated by lines(target), their individual priority is reduced.

Comparisons were conducted using the nonparametric Wilcoxon signed-rank test. Differences were considered statistically significant if the p-value was below 0.05 or approached significance within the range of 0.05 ≤ *p* < 0.1. Statistical tests were performed using XLSTAT v2022.

**Table 2:** Parameters for normal tissue objectives, dose–volume constraints for the PTV, and the OAR used to optimize the various RapidPlan™ models.

Normal Tissue Objective (NTO) parameters			
Fall-off	Distance from target Border (cm)	Start Dose / End Dose	Priority
0.3	0.2	100% / 50%	150
**Structures constraints and objectives**			
**Type**	**Volume (%)**	**Dose (% or Gy)**	**Priority**
***PTV***			
Upper	0	102%	150
Lower	100	99%	150
***Anal Canal***			
Line (target)	Generated	Generated	40
***Peritoneal cavity***			
Upper	Generated	15 Gy	150
Mean	NA	Generated	150
Line (target)	Generated	Generated	Generated
***Rectum***			
Upper	0	100%	120
Mean	NA	Generated	150
Line (target)	Generated	Generated	Generated
***Right femoral head***			
Line (target)	Generated	Generated	40
***Left femoral head***			
Line (target)	Generated	Generated	40
***Bladder***			
Upper	0	100%	120
Line (target)	Generated	Generated	40

## Results

### Evaluation of the different RP models

Table 3 Presents the training statistics for the models evaluated, with a focus on the rectum, bladder, peritoneal cavity, anal canal, and right and left femoral heads. The R^2^ and χ² values indicated similar levels of quality in the fit between the geometric and dosimetric parameters across all the models, thus ensuring comparability. Table 3 also includes the number of plans used to train each OAR and model.

**Table 3:** Regression model parameters (R^2^ and χ^2^) and the number of structures included in the different RapidPlan™ models.

RP Models	RP_46	RP_30	RP_46 + 30	RP_Seq	RP_SIB	RP_UNI
Structures	*R*²					
Rectum	0.57	0.49	0.66	0.62	0.54	0.54
Bladder	0.78	0.76	0.88	0.84	0.84	0.84
Peritoneal cavity	0.90	0.93	0.96	0.96	0.94	0.96
Anal Canal	0.86	0.69	0.83	0.80	0.71	0.74
Left femoral head	0.72	0.63	0.74	0.69	0.57	0.67
Right femoral head	0.63	0.67	0.73	0.72	0.65	0.64
**Structures**	**χ²**					
Rectum	1.08	1.08	1.03	1.02	1.06	1.01
Bladder	1.06	1.05	1.02	1.00	1.03	1.00
Peritoneal cavity	1.02	1.06	1.00	1.01	1.00	1.01
Anal Canal	1.06	1.02	1.04	1.03	1.05	1.01
Left femoral head	1.04	1.05	1.03	1.02	1.03	1.01
Right femoral head	1.02	1.05	1.03	1.02	1.02	1.02
**Structures**	**Trained structures**					
Rectum	73	84	157	195	77	272
Bladder	73	84	157	195	77	272
Peritoneal cavity	73	84	157	193	73	265
Anal Canal	73	84	158	196	77	265
Left femoral head	70	81	151	188	72	263
Right femoral head	70	81	151	189	74	260

**Fig. 2:**
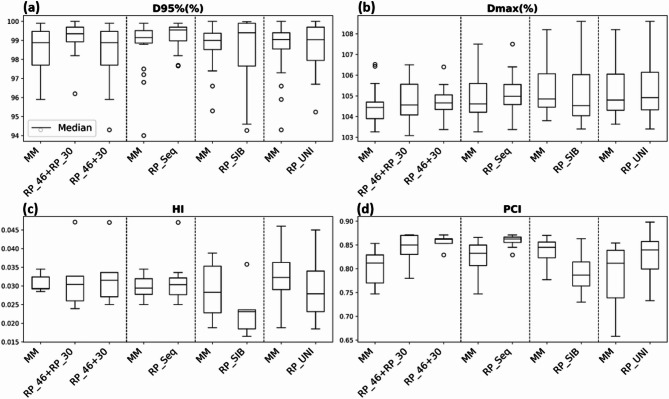
Boxplots illustrating (**a**) D95% (%), (**b**) Dmax (%), (**c**) HI, and (**d**) PCI for each model compared with the manual method.

### Comparison of the MM and rp_models

#### PTV coverage

The dosimetric parameters D95%(%), Dmax (%), HI, and PCI were used to evaluate PTV coverage. The mean (SD) D95% values (%) for the (RP_46 + RP_30), RP_46 + 30, RP_Seq, RP_SIB, and RP_UNI models were 99.2% (0.7), 99.4% (0.4), 99.2% (0.7), 98.5% (1.8), and 98.8% (1.2), respectively. The corresponding Dmax values were 104.7% (1.0), 104.7% (0.8), 105.5% (0.9), 105.1% (1.7), and 105.3% (1.5). The lowest PCI value of 0.79 (0.1) was observed for the RP_SIB model, although it remained above the threshold of 0.7. The HIs for all the models were close to 0. Figure 2 provides a detailed visualization of these results through box plots.

#### Sparing of OAR

Table 4 compares the relative differences (%) in OAR doses between manual planning and the plans generated using the RP models. Statistically significant p-values are shown in bold and underlined when they are below 0.05 or only in bold if a trend toward significance is observed (0.05 ≤ *p* < 0.1). The mean OAR dosimetric parameters for the RP plans are also shown in square brackets. Differences were observed for V45Gy(cc) in the peritoneal cavity, with the impact of the RP model varying by case. The (RP_46 + RP30), RP_46 + 30, and RP_Seq models exhibited deteriorations of 10.6% (*p* = 0.07), 12.5% (*p* = 0.06), and 16.5% (*p* = 0.06), respectively. Conversely, the RP_SIB and RP_UNI models decreased the volume receiving 45 Gy in the peritoneal cavity by 49.4% (*p* = 0.098) and 18.3% (*p* = 0.034), respectively. The RP_SIB model reduced V45Gy (cc) to 18.3 cm^3^ compared with the 36.1 cm^3^ achieved with the MM. Similarly, the RP_UNI model reduced the volume to an average of 29.2 cm^3^ versus 35.8 cm^3^ with the MM. For this dosimetric parameter, only the difference observed for the RP_UNI model was significant (*p* = 0.034). The V15Gy(cc) volume for this organ was significantly improved by approximately 18% with both the RP_SIB (*p* = 0.02) and RP_UNI (0.004) models.

The dose distributions for the rectum and anal canal were largely equivalent between the manual planning and RP models. An 8.0% (*p* = 0.036) increase in V60Gy (%) to the bladder was observed with the RP_SIB method. However, the mean V60Gy (%) to the bladder in this model was 14.1%, which was well below the clinical recommendations.

The mean doses (Dmean) to the femoral heads for the (RP_46 + RP30), RP_46 + 30, and RP_Seq models were 7–17% lower than those obtained through manual planning.

**Table 4:** Average dosimetric differences between clinical plans and plans generated by various rapidplan models. Statistically significant p-values are shown in bold and underlined when they are below 0.05 or only in bold if a trend toward significance is observed (0.05 ≤ *p* < 0.1). The mean OAR dosimetric parameters for the RP plans are also shown in square brackets.

OAR	Clinical goal	MM – (RP_46 + RP_30)	MM – RP_46 + 30	MM – RP_Seq	MM – RP_SIB		MM – RP_UNI
		Relative Difference in % (*p*-value) [Mean values for OAR dosimetric parameters for RP plans]					
***Peritoneal cavity***	Dmax(Gy)	**-4.15(0.055)** [51.5]	**-4.72(0.08)** [51.7]	-2.09(0.116) [44.5]	1.59(0.426) [50.7]		-3.75(0.145) [47.8]
	V45Gy < 150 cc	**-10.64(0.074)** [61.7]	**-12.5(0.06)** [62.8]	**-16.54(0.062)** [52.0]	**49.38(0.098)** [18.3]		**18.34(0.034)** [29.2]
	V15Gy < 830 cc	2.3(0.303) [693.4]	3.9(0.28) [682.0]	0.63(0.355) [568.4]	**18.78(0.02)** [691.1]		**17.45(0.004)** [507.0]
***Rectum***	Dmax(Gy)	**-1.51(0.001)** [77.8]	-1.21(0.51) [77.6]	**-1.51(< 0.0001)** [73.4]	**-2.06(0.01)** [72.9]		**-1.7(0.006)** [72.4]
	V60Gy < 50%	-0.15(0.105) [16.0]	-0.77(0.76) [16.6]	0.35(0.568) [16.0]	**5.4(0.002)** [7.9]		**4.22(0.001)** [9.2]
	V70Gy < 25%	0.6(0.211) [7.6]	0.90(0.13) [7.3]	0.42(0.379) [4.5]	**2.34 (0.028)** [2.2]		**1.43(0.025)** [2.4]
	V75Gy < 5%	**-1.4(0.026)** [2.9]	-1.57(1.00) [2.3]	**− 0.82(0.028)** [1.8]	1.8(1.00) [0.9]		0.02( 0.953) [1.1]
***Bladder***	Dmax(Gy)	0.0(0.421) [78.4]	0.19(0.49) [78.2]	-0.36(0.367) [74.0]	**-1.11(0.064)** [74.5]		**-0.8(0.036)** [73.7]
	V60Gy < 50%	-0.8(0.233) [12.4]	-1.74(0.169) [13.3]	**-1.85(0.007)** [17.5]	**-8.0 (0.012)** [14.1]		**-1.05(0.003)** [14.6]
	V70Gy < 25%	-0.14(0.148) [6.7]	-0.07(0.30) [6.6]	-0.06(0.460) [4.0]	-2.4(0.249) [3.6]		**-0.38(0.099)** [3.6]
***Anal Canal***	Dmax(Gy)	-1.93(0.762) [61.9]	-3.03(0.49) [62.3]	-3.71(0.210) [62.8]	5.19(0.322) [58.5]		4.24(0.355) [58.7]
***Right femoral head***	Dmean(Gy)	**16.89(0.004)** [17.0]	**10.3(0.01)** [18.3]	**12.15(0.003)** [17.3]	0.12(0.770) [17.3]		7.75(0.182) [17.2]
***Left femoral head***	Dmean(Gy)	**13.28(0.005)** [16.1]	**7.5(0.05)** [17.2]	**8.72(0.004)** [16.5]	0.18(1.0) [16.4]		2.99(0.723) [16.6]

#### Time, EPID QC and MUs for the 20 MM vs. RP_UNI patients

Dosimetry completion times were recorded manually from the creation of optimization volumes to the final clinical validation of the plan. The time measurements for RP_UNI started with the optimization process and ended with the final dose calculation. The recorded time included the summation of all plans for prescriptions containing multiple plans. The mean (SD) completion times were 129.1 (69.2) min for the MM and 44.2 (22.0) min for the RP_UNI method, resulting in an average time reduction of 85 min (p < < 0.05), equivalent to a 66% decrease.

Pretreatment quality control (QC) was conducted for all 20 patients using an onboard electronic portal imaging device (EPID) detector. The average γ-index pass rates for the MM and RP_UNI method, which use 3%/3 mm threshold criteria with a dose threshold of 5%, were 99.8% ± 0.2 and 99.7% ± 0.3%, respectively, meeting all the clinical criteria.

The number of MUs per plan obtained by the RP_UNI model compared to the manual method was 737.3 ± 186.6 vs. 674.3 ± 182.9 respectively. This average increase of 63.0 MUs per plan was significant (*p* = 0.01).

## Discussion

This study demonstrated the ability of RP methods to enhance the quality of treatment plans, particularly for complex target volumes such as the prostate, with or without seminal vesicles and pelvic lymph nodes. Unlike other studies that stratified plans based on the complexity of the target volumes, this study demonstrated the feasibility of a unified RP model accommodating all clinical cases, minimizing the need for multiple models.

Cagni et al.^26^ created two different models. The first was designed for low-risk prostate cancer (35 plans) with a delivery of 70 Gy in 28 fractions to the prostate. The second model targeted intermediate-risk cancers and included 30 SIB plans, delivering 70 Gy to the prostate and 56 Gy to the seminal vesicles in 28 fractions.

van Gysen et al.^27^ developed eight models exclusively for prostate treatment, tailored to distinguish different clinical cases. The number of plans used for training ranged from 20 to 50 per model. However, achieving clinically acceptable plans often requires up to three reoptimizations.

These examples underscore the challenges in defining the scope of clinical cases for model training and determining the number of plans necessary for implementing such tools. Centers tend to create configurations tailored to specific prescriptions.

This study proposes a methodology that uses several specific models to create a unified model, RP_UNI, applicable to all prostate cancer prescriptions (Table 1: P1–P11), including prostate bed cases.

Each step was validated through tests involving 10–25 patients external to the models and comparisons with results from manual planning. All the models produced treatment plans that complied with clinical requirements (see Table 4). The overall analysis highlighted significant sparing of the peritoneal cavity (V45Gy (cc) and V15Gy (cc)) achieved with RP_UNI model. Consequently, the RP_UNI model is the optimal choice for clinical implementation.

This approach demonstrates the feasibility of consolidating diverse clinical cases into a single RP model, surpassing the limitations of models described in the literature. The unified model accommodated both sequential and SIB techniques, enabling the inclusion of prostate volume alone or combined with seminal vesicles, with or without pelvic lymph nodes. Additionally, the integration of prostate bed cases presented no complications. The RP_UNI model was developed with 272 training plans using this methodology: 195 plans employing the sequential boost technique (Table 1: P1–P5) and 77 plans using the SIB technique (Table 1: P6–P11).

The significant time saving achieved through the Varian automated solution was confirmed, with an average reduction of 85 min (p < < 0.05) compared with the manual method.

Pretreatment QC using the portal imager validated the clinical deliverability of RP_UNI treatment plans with an average gamma index of 99.7% ± 0.3% for 3%/3 mm criteria.

We also observed a significant mean increase of 63.0 MUs per plans for the RP_UNI model compared with the manual method. This trend had already been identified in a study by Wall and Fontenot^28^, but we considered it acceptable when taking into account the results of the pretreatment QC. The development and implementation of the RP solution using this step-by-step methodology required considerable effort. Two experienced physicists, supported by a medical physics trainee, worked for more than four months to produce the RP_UNI model for clinical use. Ensuring equivalent statistical quality across the compared models was critical (Table 3). The analysis of dosimetric, geometric, and overfitting outliers adhered not only to the manufacturer’s guidelines but also to established studies by Delaney et al.^21^ and Alpuche Aviles et al.^22^. The configuration of the model effectively requires the operators in charge of setting it up to be fully proficient with the tool. They must imperatively follow the training proposed by the manufacturer.

One of the goals of these automated tools is to reduce the learning curve for any new operator. This is especially true since no operator intervention was allowed in this retrospective study. However, we recommend performing manual dosimetry for the first patients, then running the RP solution for comparison. Above all, this method allows the operator to gain confidence in the tool and to acquire a critical view of its use.

Preliminary testing determined the optimal constraints and priority settings for the target volumes and OARs within the models (Table 2). These results fall outside the scope of the current study. For example, the “generated” option of the RP system, which automatically assigns weights and priorities, failed to adequately differentiate between major OARs (rectum, bladder, and peritoneal cavity) with challenging objectives and minor OARs (e.g., femoral heads) that are easier to spare. Consequently, high-priority values were manually assigned to major OARs, whereas lower values were applied to minor OARs.

These final considerations remain highly dependent on the specific center despite their notable influence on the treatment plans generated by KBP models^22^.

Although we have proposed models that support the majority of our patients, special cases such as hip replacements were not taken into account during the RP modeling phase. These cases may have an impact on the quality of the resulting treatment plans. The validation steps by the physicist and the physician remain crucial.

The selection of training plans for the RP models did not consider the type of accelerator used in patient treatment. This approach aligns with the findings in the literature. For example, Costa et al.^29^ demonstrated that a model trained on a conventional linear accelerator using 6MV FF can be effectively applied to a Halcyon© accelerator operating with 6MV FFF. This finding has enabled us to consider our equipment park, two Halcyon© units and one Truebeam© system, for patient management. Our study is primarily aimed at users of the RapidPlan™ solution. Comparing the dosimetric or time performance with other existing commercial solutions would also be an interesting study that was not carried out within the scope of this work. Smith et al.^30^ in 2019, compared RP and Pinnacle Auto-Planning without finding significant dosimetric differences.

The statistical parameters proposed by RP, such as R^2^ and χ², proved insufficient for reliably predicting model efficiency (Table 3). The outcomes of the resulting treatment plan varied significantly despite the use of equivalent statistical parameters for different models. A systematic approach, such as the one adopted in this study, is essential to ensure optimal model performance.

The next phase of this study involved extending the systematic approach to other tumor types, particularly breast, head, and neck cancers, which present clinical diversity challenges identical to those observed in prostate cancer. Having a single model for all prescriptions for a given site is simpler and more reassuring for users than having a multitude of models that only deal with specific cases.

The proposed model was validated exclusively using data from our center. Nevertheless, recent studies have explored the potential for sharing a single RP model across multiple institutions^31–33^. For example, Schubert et al.^31^ evaluated the performance of an RP model developed at one center and applied across seven other centers within the German RP consortium. This model, trained on data from 43 patients treated for prostate and seminal vesicle cancers with pelvic lymph nodes in a sequential boost, produced dosimetry results that fully adhered to clinical requirements. Conversely, Fukunaga et al.^33^ recommended the use of large-scale models trained on data from over 500 patients to improve dosimetric quality. However, their study focused exclusively on prostate and seminal vesicle treatments.

Therefore, another area of investigation would be to assess the performance of the RP_UNI unified model using plans from external institutions. This ongoing study aims to validate the model under varying prescriptions and delineation protocols, based on these initial mono-centric results.

Furthermore, longitudinal studies assessing the long-term clinical outcomes of patients treated with KBP-guided plans could provide valuable insights into the sustained benefits of this approach. While dosimetric results are promising, no prospective study has validated whether these improvements translate into better therapeutic outcomes or reduced toxicity rates.

## Conclusions

This study successfully developed a unified RP model encompassing all 11 medical prescriptions for prostate cancer treatment at our center. The model achieved clinical effectiveness comparable to or superior to that of the manual method while significantly reducing the planning time. These outcomes provide a foundation for extending this methodology to other tumor sites.

## Data Availability

The datasets used and/or analysed during the current study are available from the corresponding author on reasonable request.

## Abbreviations

RP: RapidPlan^™^
KBP: Knowledge-based planning
IMRT: Intensity-modulated radiation therapy
PTV: planning target volume
VMAT: volumetric modulated arc therapy
OAR: Organ at risk
DVH: Dose–volume histogram
HI: Homogeneity index
QC: Quality control
EPID: Electronic portal imaging device
FF: Flattening filter
FFF: Free flattening filter
TPS: Treatment planning system
SIB: Simultaneous integrated boost
PCI: Paddick conformity index
